# miR-199a-3p targets stemness-related and mitogenic signaling pathways to suppress the expansion and tumorigenic capabilities of prostate cancer stem cells

**DOI:** 10.18632/oncotarget.10652

**Published:** 2016-07-18

**Authors:** Ruifang Liu, Can Liu, Dingxiao Zhang, Bigang Liu, Xin Chen, Kiera Rycaj, Collene Jeter, Tammy Calhoun-Davis, Yandong Li, Tao Yang, Junchen Wang, Dean G. Tang

**Affiliations:** ^1^ Cancer Stem Cell Institute, Research Center for Translational Medicine, East Hospital, Tongji University School of Medicine, Shanghai 200120, China; ^2^ Department of Epigenetics and Molecular Carcinogenesis, The University of Texas MD Anderson Cancer Center, Science Park, Smithville, TX 78957, USA; ^3^ Department of Pharmacology & Therapeutics, Roswell Park Cancer Institute, Buffalo, NY 14263, USA; ^4^ Centers for Cancer Epigenetics, Stem Cell and Developmental Biology, RNA Interference and Non-coding RNAs, and Molecular Carcinogenesis, The University of Texas MD Anderson Cancer Center, Houston, TX 77030, USA

**Keywords:** prostate cancer, miR-199a-3p, CD44, c-MYC, cancer stem cells

## Abstract

Human cancers exhibit significant cellular heterogeneity featuring tumorigenic cancer stem cells (CSCs) in addition to more differentiated progeny with limited tumor-initiating capabilities. Recent studies suggest that microRNAs (miRNAs) regulate CSCs and tumor development. A previous library screening for differential miRNA expression in CD44^+^ (and other) prostate CSC vs. non-CSC populations identified miR-199a-3p to be among the most highly under-expressed miRNAs in CSCs. In this study, we characterized the biological functions of miR-199a-3p in CD44^+^ prostate cancer (PCa) cells and in tumor regeneration. Overexpression of miR-199a-3p in purified CD44^+^ or bulk PCa cells, including primary PCa, inhibited proliferation and clonal expansion without inducing apoptosis. miR-199a-3p overexpression also diminished tumor-initiating capacities of CD44^+^ PCa cells as well as tumor regeneration from bulk PCa cells. Importantly, inducible miR-199a-3p expression in pre-established prostate tumors in NOD/SCID mice inhibited tumor growth. Using target prediction program and luciferase assays, we show mechanistically that CD44 is a direct functional target of miR-199a-3p in PCa cells. Moreover, miR-199a-3p also directly or indirectly targeted several additional mitogenic molecules, including c-MYC, cyclin D1 (CCND1) and EGFR. Taken together, our results demonstrate how the aberrant loss of a miRNA-mediated mechanism can lead to the expansion and tumorigenic activity of prostate CSCs, further supporting the development and implementation of miRNA mimics for cancer treatment.

## INTRODUCTION

Human cancers are heterogeneous containing phenotypically differentiated cancer cells as well as immature stem-like cancer cells or cancer stem cells (CSCs) [1, 2]. CD44, a cell surface adhesion receptor with pleiotropic signaling functions, is highly enriched in and has been used to enrich CSCs in a variety of tumors [3–5]. Systematic studies from our lab have demonstrated that CD44 is a prostate cancer stem cell (PCSC) enrichment marker that plays a causal role in prostate cancer (PCa) development and metastasis [6–10]. For example, purified CD44^+^ cell population demonstrates high tumorigenic and metastatic potential [6] and knockdown of CD44 inhibits tumorigenicity and metastasis of PCa cells in multiple models [9]. Also, CD44^+^ cells are relatively quiescent, express high levels of “stemness” genes including Oct-3/4, Bmi, β-catenin and SMO [6]. PCa cells double-positive for CD44 and integrin α2β1 (i.e., CD44^+^α2β1^+^) are even more tumorigenic than CD44^+^ PCa cells [7]. Finally, we have shown that the CD44^+^α2β1^+^ALDH^+^ subpopulation in the undifferentiated (PSA^−/lo^) cell pool identifies highly tumorigenic and castration-resistant PCa cells [8]. Together, these studies highlight the involvement of CD44^+^ PCSCs in PCa development, metastasis and therapy resistance and suggest that it will be important to understand how PCSCs are molecularly regulated.

MicroRNAs (miRNAs), ~22nt small non-coding RNAs, exert their functions via base-pairing with the target mRNA. Over 60% of human coding genes contain at least one conserved miRNA binding site, and most coding genes in the human genome are probably under the control of miRNAs [11]. Dysregulation of miRNA expression and functions has been widely reported and some miRNAs have been explored as anti-cancer therapeutics [12]. Nevertheless, miRNA regulation of CSCs in general and PCSCs in particular remains incompletely understood. Recent evidence suggests that miRNAs may play important functions in regulating CSCs and tumor development [13, 14]. Our earlier miRNA library screening has identified several miRNAs, i.e., miR-34a, let-7b, miR-141, and miR-106 that are commonly under-expressed in tumorigenic PCa cell subsets including CD44^+^, CD133^+^ and α2β1^+^ PCa cells [9, 15]. Functional interrogations on miR-34a [9] and let-7a [15] revealed prostate tumor- and/or metastasis-suppressive functions for the two miRNAs, which function via different mechanisms. miR-34a is the first miRNA being developed for cancer therapy and is currently in a phase II clinical trial for primary liver cancer [16]. Interestingly, miR-199a-3p is one of the miRNAs most dramatically underexpressed in the CD44^+^ PCa cell populations uncovered in our miRNA library screening [15].

MiR-199a-3p is an under-studied miRNA, especially in PCa, with only one report so far showing miR-199a-3p underexpression in PCa compared to benign tissues [17]. In this study, we present evidence for tumor suppressive functions of miR-199a-3p in both purified CD44^+^ and bulk PCa cells based on *in vitro* clonogenic and *in vivo* tumor regeneration assays as well as therapeutic experiments. We also show that miR-199a-3p exerts its PCa suppressive functions via targeting CD44 and several mitogenic molecules including c-MYC, cyclin D1 and EGFR.

## RESULTS AND DISCUSSION

### miR-199a-3p inhibits PCa cell proliferation *in vitro*

**Figure 1:**
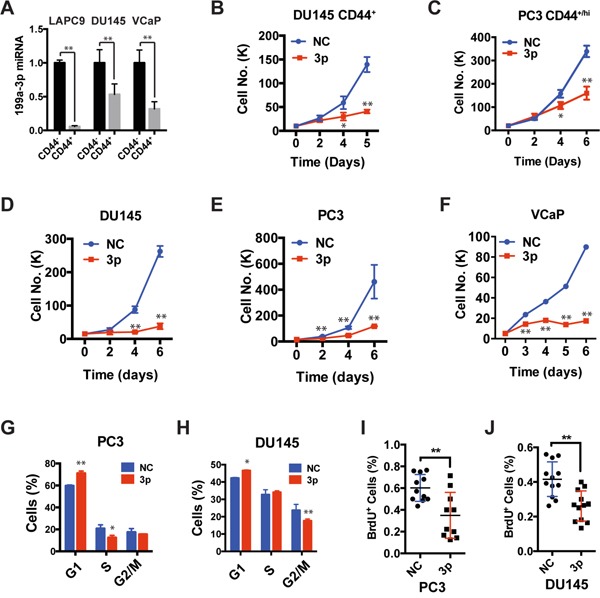
Expression of miR-199a-3p inhibits cell proliferation **A.** Relative expression levels of miR-199a-3p. CD44^+^ and CD44^−^ cells were purified from DU145 cultures and two xenografts (LAPC9 and VCaP) and total RNA was used in qPCR. The y-axis represents the miR-199a-3p levels in CD44^+^ cell population relative to its levels in CD44^−^ population. **B-F.** Cell viability assays. CD44^+^ DU145 (B) and PC3 (C) cells, or bulk DU145 (D), PC3 (E), and VCaP cells (F) were transfected with 30 nM of NC or miR-199a-3p oligos and plated (20,000 cells/well) at day 0 and live cells counted at indicated days under microscope. **G-H.** DNA content analysis in bulk PC3 (G) or DU145 (H) cells transfected with miR-199a-3p or NC (30 nM, 48 h). Bars represent the average percentage of cells in each cell-cycle phase. **I-J.** BrdU incorporation assays in PC3 and DU145 cells transfected with miR-199a-3p or NC (30 nM, 72 h). All bars and data points represent the mean ± S.D from 2-5 independent experiments with each condition having 2-3 replicates in each experiment. *P<0.05, **P<0.01.

miR-199a-3p has been reported as a tumor suppressive miRNA in several tumor types. Most miR-199a-3p related studies are in hepatocellular carcinoma (HCC), in which it is reported to induce apoptosis or to suppress cell proliferation by delaying G1/S transition [18–20]. Overexpression of miR-199a-3p has also been reported to result in caspase-dependent and -independent apoptosis in lung cancer [21] and G1 phase cell-cycle arrest in osteosarcoma cells [22]. Our previous study suggested that miR-199a-3p is underexpresssed in several PCa stem/progenitor cell populations, especially in CD44^+^ PCa cells [9, 15]. In the present study, we started by re-evaluating miR-199a-3p expression in the CD44^+^ cell population, freshly purified from DU145 cultures and two xenografts, i.e., LAPC9 and VCaP. The results revealed significant under-expression of miR-199a-3p in all three CD44^+^ PCa cell populations (Figure 1A). In the forgoing sections, we set out to determine the biological functions of miR-199a-3p in two AR^+^/PSA^+^ (i.e., LAPC9 and VCaP) and three AR^−^/PSA^−^ PCa cell line (DU145, PC3, and PPC-1) and xenograft (LAPC9 and VCaP) models. In these 5 PCa models, 3 (i.e., LAPC9, VCaP, and DU145) have well-demarcated CD44^+^ and CD44^−^ subpopulations whereas PC3 and PPC-1 cells are nearly 100% positive although CD44^+/hi^ and CD44^−/lo^ subpopulations could still be fractionated out [6, 9, 10].

We transfected miR-199a-3p mimics or negative control (NC) oligos into either purified CD44^+^ (Figure 1B-1C) or bulk (Figure 1D-1F) PCa cells. miR-199a-3p reduced the live cell numbers in both purified CD44^+^ (Figure 1B-1C) and bulk (Figure 1D-1F) PCa cells. To uncover the potential mechanisms underlying the PCa cell “growth-inhibitory” effects of miR-199a-3p, we assessed cell proliferation by BrdU incorporation and cell-cycle (i.e., DNA content) analysis, cell death by Annexin V and PI staining, and cell senescence by senescence-associated β-galactosidase staining. We observed that miR-199a-3p treatment increased the % of G1-phase cells in PC3, DU145, and PPC-1 cultures (Figure 1G and 1H; Supplementary Figure 1B-1D). For example, in PC3 cells, the G1-phase cells increased from ~60% in the NC group to ~67% in the miR-199a-3p group (Figure 1G; Supplementary Figure 1D). Interestingly, accompanying the increase in G1-phase cells, miR-199a-3p reduced S-phase cells in PC3 (Figure 1G; Supplementary Figure 1D) but reduced G2/M-phase cells in DU145 (Figure 1H; Supplementary Figure 1D) and PPC-1 (Supplementary Figure 1B; Supplementary Figure 1D) cells. These results suggest that in 3 PCa cell types, miR-199a-3p overexpression causes G1 cell-cycle arrest with concomitant decrease in S or G2/M phase cells. Consistent with the cell-cycle analysis, miR-199a-3p inhibited BrdU incorporation in PC3 (Figure 1I) and DU145 (Figure 1J) cells. In contrast, no significant difference was observed between NC and miR-199a-3p treated PCa cells in early apoptotic, late apoptotic or late necrotic cells (Supplementary Figure 1E). Neither miR-199a-3p nor NC induced appreciable cell senescence in the 3 PCa cell types (date not shown). Taken together, these observations indicate that enforced expression of miR-199a-3p inhibits PCa cell cell-cycle progression and proliferation without affecting cell death or senescence.

### miR-199a-3p inhibits PCSC properties

**Figure 2:**
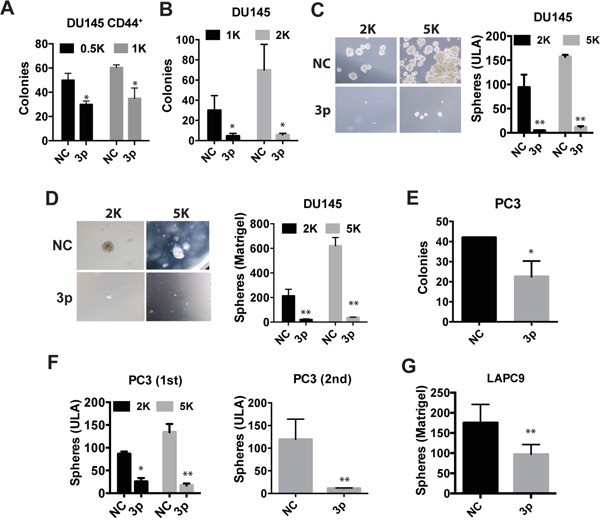
miR-199a-3p suppresses clonogenic and sphere-forming properties in PCa cells **A-B.** Holoclone assays in freshly purified CD44^+^ (A) or bulk DU145 (B) cells. NC or miR-199a-3p transfected cells (numbers indicated) were plated in 6-well plates and holoclones enumerated on day 12 (for A) and 14 (for B), respectively. **C-D.** Sphere assays in bulk DU145 cells. Cells were plated on 6-well ULA plates (C) or mixed with Matrigel (1:1) before plating (D) and spheres scored on day 13. **E-G.** Holoclone and sphere-formation assays in bulk PC3 (E, F) and LAPC9 (G) cells transfected with NC or miR-199a-3p. For E, 100 cells were plated and holoclones scored on day 15. For F, primary sphere assays were conducted by plating 2K or 5K cells in ULA plates and spheres scored on day 7. In secondary sphere assays, the first generation spheres were harvested, digested into single cells, and replated at 5K cells, and spheres scored on day 25. For G, 5K cells were plated and spheres scored on day 18. Cells were transfected with NC or miR-199a-3p oligos at 30 nM. All bars represent the mean ± S.D from 2-4 independent experiments with each condition having 3 replicates. *P<0.05, **P<0.01.

Kinose et al reported that miR-199a-3p was downregulated under hypoxia and decreased the clonal capacity in ovarian cancer cells [23]. PCa cell holoclones contain self-renewing tumor-initiating cells [24] and spheres formed under anchorage-independent conditions harbor tumor-initiating cells [6, 9, 25]. To test the effects of miR-199a-3p on PCSC properties, we employed holoclone, Matrigel-based clonogenic, and ultra-low attachment (ULA) based sphere-formation assays (Figure 2), which have been widely used to measure the activity of stem/progenitor cells. Purified CD44^+^ DU145 cells transfected with miR-199a-3p oligos exhibited significantly reduced cloning efficiency compared with the cells transected with NC oligos (Figure 2A). Bulk DU145 cells were also dramatically suppressed by miR-199a-3p in all of the abovementioned three assays (Figure 2B-2D). miR-199a-3p showed similar inhibitory effects in PC3 and LACP9 cells (Figure 2E-2G). Notably, miR-199a-3p inhibited secondary sphere formation in PC3 cells (Figure 2F), suggesting that miR-199a-3p may inhibit PCSC self-renewal *in vitro*. Collectively, these observations demonstrate that miR-199a-3p negatively regulate PCSC properties.

### miR-199a-3p demonstrates inhibitory effects in primary human PCa (HPCa) cells

**Figure 3:**
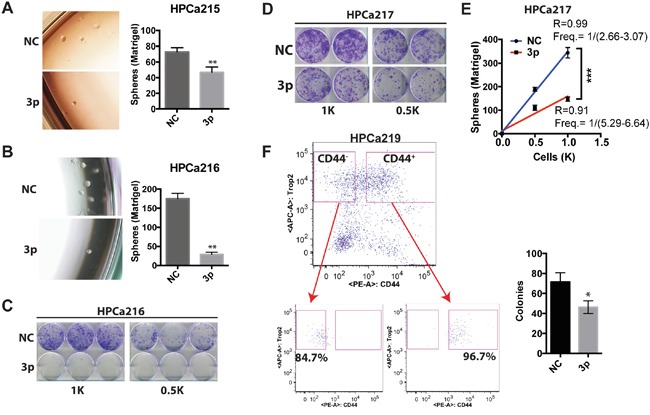
miR-199a-3p inhibits clonal and clonogenic properties of HPCa cells **A.** Matrigel-based sphere assays in HPCa 215 cells. 400 cells were plated in triplicate in six-well plate and spheres scored on day 7. **B-C.** Matrigel-based sphere (B) and holoclone (C) assays in HPCa216 cells. For B, 500 cells were plated in triplicate and spheres scored on day 7. For C, the indicated number of cells was plated in six-well plate and images taken on day 8. **D-E.** Holoclone (D) and Matrigel-based sphere (E) assays in HPCa217 cells. For D, the indicated numbers of cells were plated in six-well plate and images taken on day 8. For E (limiting dilution sphere assays), 500 or 1,000 cells were plated in six-well plates and colonies scored on day 8. **F.** Holoclone assays in CD44^+^ cells sorted from HPCa219 patient tumor. 400 TROP2^+^CD44^+^ cells (purity 96.7%, below) were plated in triplicate and holoclones scored on day 9. Cells transfected with NC or miR-199a-3p (3p) oligos at 30 nM were used in above experiments (n=2-3 for each experiment). All bars and data points represent the mean ± S.D; *P<0.05, **P<0.01.

The miR-199a-3p expression level is generally decreased in cancers in comparison to their normal counterparts [18, 20, 22, 26]. In PCa, miR-199a-3p expression is found to be negatively associated with tumor staging and differentiation [17]. However, very few functional studies have been performed in human primary cancer samples. Consequently, we studied the biological functions of miR-199a-3p in 4 HPCa specimens with ~100% tumor involvement (Supplementary Figure 2A-2D). Tumor pieces were quickly processed and epithelial HPCa cells were purified out (see Materials & Methods) and transfected with miR-199a-3p or NC oligos. Bulk HPCa cells with miR-199a-3p overexpression demonstrated much lower sphere-forming (Figure 3A-3B) and clonal (Figure 3C-3D) capacities than the corresponding HPCa cells transfected with NC oligos. We also performed a limiting dilution sphere formation assay in HPCa217 cells and the results demonstrated that miR-199a significantly reduced the sphere-forming activities (Figure 3E). Finally, we purified out the CD44^+^/CD44^−^ HPCa219 epithelial cells (i.e., using the TROP2 as the epithelial marker [27] (Figure 3F, left; purities for each population shown below) and performed clonal analysis. miR-199a-3p overexpression significantly reduced colony formation of the CD44^+^ HPCa219 cells (Figure 3F, right). As we observed before that the CD44^−^ HPCa cells generally manifest low/no clonal capacity [10], the CD44^−^ HPCa219 cells hardly formed any holoclones (data not shown). These results indicate that miR-199a-3p also manifests inhibitory effects in primary PCa cells.

### miR-199a-3p suppresses prostate tumor regeneration *in vivo*

**Figure 4:**
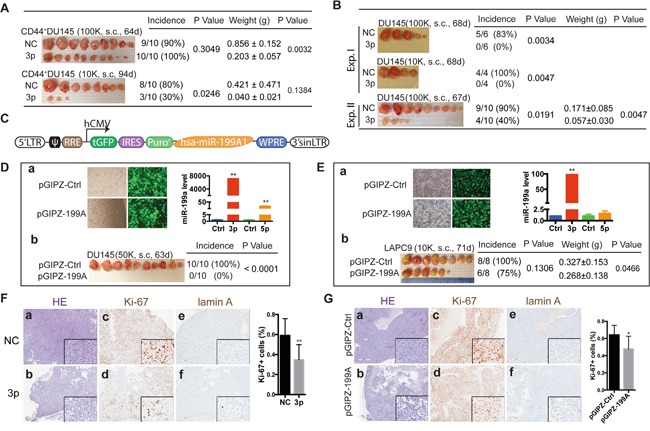
miR-199a-3p inhibits xenograft tumor regeneration **A.** Tumor regeneration assays in purified CD44^+^ DU145 cells, transfected with NC or miR-199a-3p (30 nM, 48 h) and s.c. injected, at 2 cell doses, into NOD/SCID mice. Tumor harvest time, weight, incidence and the corresponding P values are indicated. **B.** Tumor regeneration assays in bulk DU145 cells transfected with NC or miR-199a-3p oligos (30 nM, 48 h) and s.c. injected in two independent experiments. **C.** Schematic showing miR-199a-3p expressing vector pGIPZ-199A based on GIPZ lentiviral shRNA backbone (pGIPZ-Ctrl). hsa-miR-199A1, human miR-199A1 and its flanking sequences (759 bp), inserted into XhoI and MluI sites. **D-E.** Subcutaneous tumor regeneration from DU145 (D) and LAPC9 (E) cells infected with pGIPZ-199A or pGIPZ-Ctrl lentivirus. DU145 cells were infected with the lentiviruses (MOI =10) followed by puromycin selection for ~2 weeks (D). LAPC9 cells were similarly infected for 48 h without puromycin selection (E). GFP images and bar graphs showed the transduction efficiency of pGIPZ-199A. The relative expression levels of miR-199a-3p and miR-199a-5p were measured by RT-qPCR. Shown in panels b are tumor harvest time, weight, incidence and P values. **F-G.** HE and IHC staining for tumors generated in NC or miR-199a-3p transfected CD44^+^ DU145 (F) and pGIPZ-Ctrl or pGIPZ-199A transduced LAPC9 (G) cells. 4–8 fields were chosen from each slide for counting Ki-67^+^ cells. Original magnification: 40x, insets: 400x.

miR-199a-3p has been shown to inhibit peritoneal dissemination of ovarian carcinoma cells in a xenograft model [23]. However, studies on *in vivo* functions of miR-199a-3p in human cancers are generally very limited. To determine whether miR-199a-3p possesses tumor-inhibitory effects in PCa, we carried out limiting-dilution assays (LDAs) in immunocompromised mice by monitoring tumor latency, incidence and endpoint weight. First of all, we transfected miR-199a-3p and NC oligos into freshly purified CD44^+^ DU145 cells and subcutaneously implanted them into NOD/SCID mice. As shown in Figure 4A, at 100,000 cell injections, miR-199a-3p significantly inhibited tumor growth as manifested by reduced tumor sizes. At 10,000 injections, miR-199a-3p inhibited both tumor incidence and tumor growth (Figure 4A; note that miR-199a-3p overexpressing CD44^+^DU145 cells regenerated tumors that were only 1/10 of the tumors derived from NC-transfected CD44^+^DU145 cells). Impressively, in two independent experiments, miR-199a-3p nearly completely abolished tumor regeneration from bulk DU145 cells (Figure 4B). miR-199a-3p overexpression by oligo transfection also inhibited tumor regeneration in PPC-1 and PC3 cells (data not shown).

To further investigate the tumor-inhibitory effects of miR-199a-3p, we constructed a lentiviral expression vector that encodes human miR-199A1 (Figure 4C; Supplementary Figure 1A). Consistent with our earlier observations (Supplementary Figure 1C), transduction of DU145 cells with miR-199A1 did not cause appreciable cell death but led to significantly increased amount of miR-199a-3p (Figure 4D, a). Strikingly, miR-199a-3p overexpression completely inhibited tumor regeneration from bulk DU145 cell (Figure 4D, b). We then infected bulk LAPC9 cells purified from androgen-dependent xenografts with the control or miR-199A1 encoding lentivirus for ~48 h. Again we did not observe significant cell death in LAPC9 cells infected with either virus (Figure 4E, left). pGIPZ-199A infection of LAPC9 cells for a short period of time (i.e., 48 h) led to only ~100 fold increase in miR-199a-3p levels (Figure 4E, a, right), much lower than in puromycin-selected DU145 cells (Figure 4D, a, right). Nevertheless, miR-199a-3p overexpression still reduced tumor incidence and weight in LAPC9 cells (Figure 4E, b). Note that the miR-199A1 lentivector did encode miR-199a-5p; however, the miR-199a-5p levels in both DU145 and LAPC9 cells were much lower than miR-199a-3p levels (Figure 4D-4E), suggesting that the PCa-suppressive effects we observed were largely ascribed to miR-199a-3p.

We performed HE and IHC analysis of proliferation (by Ki-67 staining) and apoptosis (by cleaved lamin A staining) in endpoint DU145 (Figure 4F) and LAPC9 (Figure 4G) tumors. In both cases, we observed, in miR-199a-3p overexpressing tumors, reduced cellularity (Figure 4F-4G; compare panels a vs. b) and Ki-67^+^ cells (Figure 4F-4G; compare panels c vs d). In contrast, both DU145 and LAPC9 tumors showed very little apoptotic (i.e., lamin A^+^) cells and there were no differences between control and miR-199a-3p tumors (Figure 4F-4G; compare panels e vs f).

Taken together, the above experiments indicate that miR-199a-3p inhibits prostate tumor regeneration and growth by inhibiting cell proliferation without causing cell death.

### miR-199a-3p exhibits therapeutic potential in a PCa xenograft models

**Figure 5:**
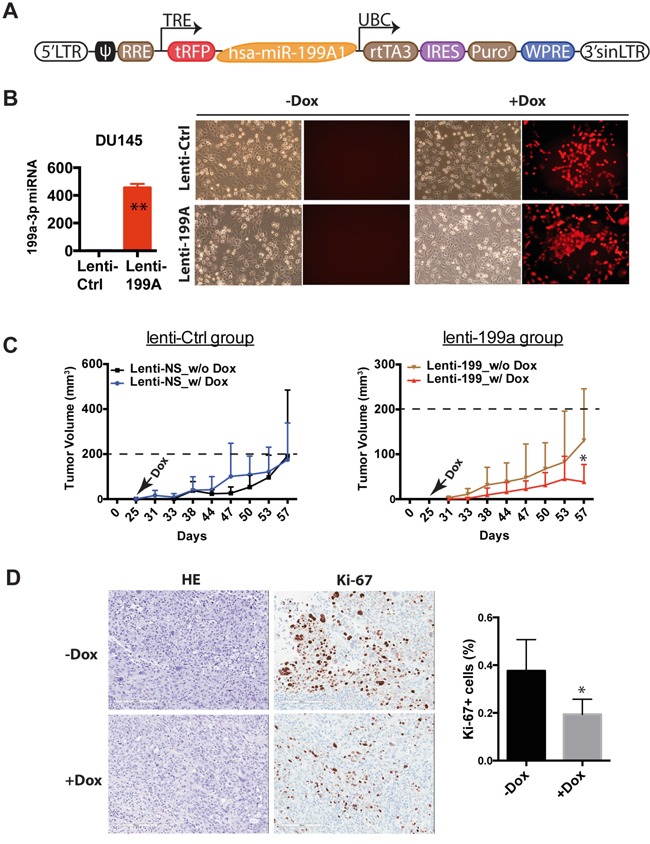
miR-199a-3p exhibits therapeutic potential in PCa cells **A.** Schematic of inducible miR-199a-3p expressing lentiviral vector. **B.** qPCR analysis of miR-199a-3p after Dox treatment for 72 h (left panel). Images show RFP expression before and after Dox administration (right panel). **C.** Measurement of tumor volume on the indicated days in lenti-199a (right) and lenti-Ctrl group (left) without or with Dox supplied in the feed on day 25. *P<0.05 between the two groups. **D.** HE and Ki-67 staining comparison between the two subgroups, i.e., before and after Dox (original magnification: 150x). Shown on the right is a bar graph presenting Ki-67^+^ cells (4 – 8 fields were counted from each slide). *P<0.05.

To explore the therapeutic potential of miR-199a-3p in PCa, we set out to test its tumor-inhibitory effects in a pre-established PCa xenograft model. To that end, we first constructed a doxycycline (Dox) inducible lentiviral system to overexpress miR-199a-3p (lenti-199a), in which primary miR-199A1 sequence was cloned downstream from the RFP reporter (Figure 5A). Dox addition induced RFP reporter expression and increased miR-199a-3p levels (Figure 5B). To perform the therapeutic experiment, we infected DU145 cells with lenti-199a or empty lenti-Ctrl vector at an MOI of 10 and implanted tumor cells subcutaneously in NOD/SCID mice. By 25 days, both lenti-Ctrl and lenti-199a groups were divided into two subgroups, one of which started to receive Dox-supplemented feed. As presented in Figure 5C (right), Dox induction in the lenti-199a group slowed down tumor growth (for unknown reasons, the lenti-199a group of tumors in the absence of Dox, without leakage of miR-199a-3p expression (data not shown)), also showed slightly slower growth compared to the corresponding lenti-Ctrl group). In contrast, the lenti-Ctrl group of tumors showed similar growth kinetics in the presence or absence of Dox (Figure 5C, left). IHC analysis again revealed reduced Ki-67^+^ cells in Dox-treated lenti-199a tumors (Figure 5D) without significant differences in apoptosis. These results, collectively, reveal a therapeutic potential of miR-199a-3p in PCa.

### CD44 is a direct target of miR-199a-3p in PCa cells

**Figure 6:**
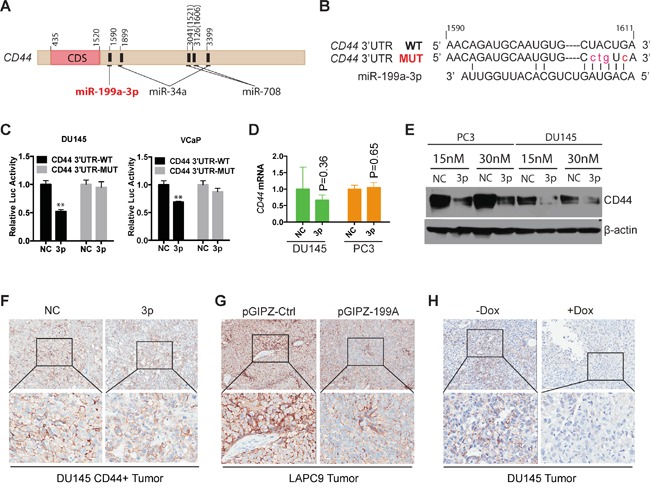
CD44 is a direct target of miR-199a-3p **A.** Schematic of the *CD44* 3′-UTR with several miRNA binding sites indicated. CD44 transcription ID: ENST00000263398. The location of 1521 and 1606 in parenthesis was reported in reference (28). **B.** Predicted duplex formed between miR-199a-3p and 3′-UTR of CD44 by the RNA22 program. Red lower case letters highlight the mutated nucleotides. **C.** Luciferase reporter assays documenting the luciferase activities in DU145 and VCaP cells co-transfected with miR-199a-3p/NC oligos with *CD44* 3′-UTR wild-type (WT) construct or the mutant (MUT). Values represent the mean ± SEM (n=4). **P<0.01. **D-E.** mRNA (D) and protein (E) of CD44 in NC or miR-199a-3p transfected DU145 and PC3 cells. **F-H.** IHC staining of CD44 in endpoint tumors. Original magnifications: top panels (80x); bottom panels (400x).

So how did miR-199a-3p exert its PCa-suppressive effects? miR-199a-3p was initially uncovered from our miRNA library screening for miRNAs differentially expressed in tumorigenic PCa cell subpopulations [9, 15]. Of interest, miR-34a was found to be underexpressed in CD44^+^ PCa cells and to inhibit PCSCs and PCa metastasis by directly targeting CD44 via binding to 2 sites at the *CD44* 3′-UTR [9] (Figure 6A). Furthermore, another miRNA, miR-708, was also reported to negatively regulate PCSC activity by targeting *CD44* at two different sites [28](Figure 6A). Finally, miR-199a-3p was reported to target CD44 in HCC cells [19]. These discussions, together with the fact that miR-199a-3p was significantly underexpressed in CD44^+^ PCa cells [15] (Figure 1A), raise the possibility that miR-199a-3p exerts its PCa-inhibitory effects via targeting, at least partly, CD44.

To test this possibility, we employed 8 different target-prediction programs, 3 of which (i.e., RNA22, TargetMiner, and TargetScan) simultaneously identified a putative binding site of miR-199a-3p at the *CD44* 3′-UTR (Figure 6A-6B). We performed site-specific mutagenesis by mutating several nucleotides at the miR-199a-3p binding site on *CD44* 3′-UTR (Figure 6B). Luciferase reporter assays in DU145 and VCaP cells showed that miR-199a-3p oligos co-transfected with the WT CD44 3′-UTR reduced luciferase activities (Figure 6C). In contrast, mutations in the miR-199a-3p binding site at *CD44* 3′-UTR abolished the luciferase-inhibitory effects of miR-199a-3p in both cell types (Figure 6C).

Interestingly, miR-199a-3p overexpression did not reduce *CD44* mRNA levels in PCa cells (Figure 6D), suggesting that miR-199a-3p likely targets CD44 in PCa cells by causing translational inhibition. Indeed, exogenously introduced miR-199a-3p reduced the CD44 protein levels in both PC3 and DU145 PCa cells (Figure 6E). Importantly, CD44 protein levels were also reduced in the endpoint tumors derived from CD44^+^DU145 cells transfected with miR-199a-3p oligos (Figure 6F), LAPC9 cells infected with the pGIPZ-199A (Figure 6G), and DU145 cells infected with the Dox-inducible lenti-199A (Figure 6H).

### Evidence that miR-199a-3p also targets c-MYC, cyclin D1, and EGFR in PCa cells

It is well-established that a single miRNA may target multiple mRNA molecules. In fact, miR-199a-3p has been shown to suppress, in addition to CD44 [19], several other molecules including MET, mTOR, and PAK4 [18, 20]. We wondered what other molecules miR-199a-3p might also target in PCa cells, either directly or indirectly. Since our preceding experiments have shown that miR-199a-3p suppressed PCa primarily by inhibiting cell-cycle progression and cell proliferation, we subsequently focused our efforts on 3 mitogenic molecules important for regulating PCa cell proliferation, i.e., c-MYC, cyclin D1, and EGFR. The *c-MYC* gene is known to be amplified and overexpressed in a variety of human tumors including PCa [29, 30] and the c-MYC protein is sufficient to immortalize benign prostatic epithelial cells [31]. c-MYC has also been shown to regulate PCSCs [32]. cyclin D1 overexpression, combined with inactivated PTEN and SMAD4 and increased SPP1, was reported to be highly predictive for poor clinical outcome in PCa [33]. The proliferation-promoting role of cyclin D1 in PCa was also corroborated in a transgenic mouse study [34]. Finally, EGFR, as an important member of the oncogenic tyrosine kinases, has been implicated in aggressive PCa [35].

**Figure 7:**
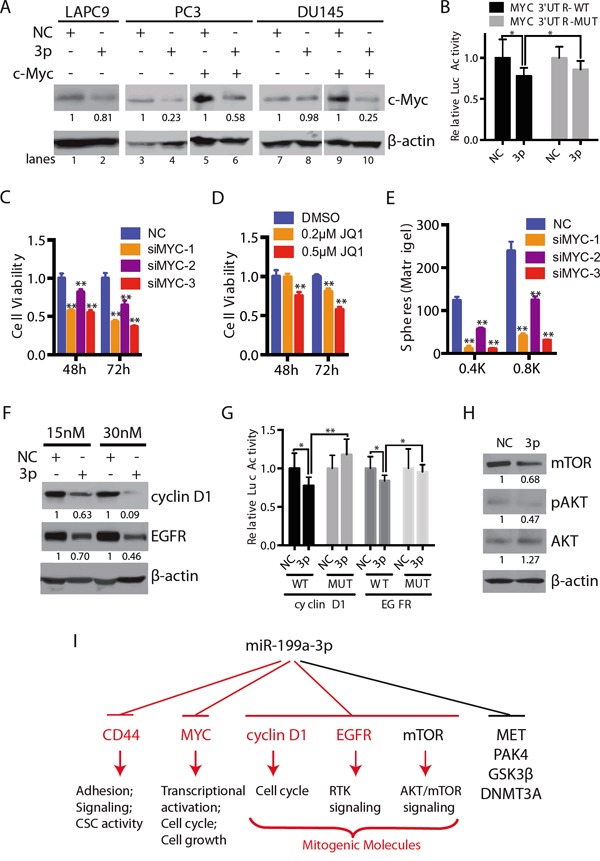
miR-199a-3p also targets c-MYC and several other mitogenic signaling molecules **A.** Western blotting (WB) showing the protein levels of c-MYC in LAPC9, PC3 and DU145 cells transfected with NC or miR-199a-3p (lanes 1-4 and 7-8) or co-transfected with pCDH-MYC vector (lanes 5-6 and 9-10) for 72 h. **B.** Luciferase assays showing the activity of WT or mutant MYC 3′UTR in PC3 cells expressing miR-199a-3p. **C-D.** Cell viability of c-MYC siRNAs (20 nM; C) or small molecule inhibitor JQ1 (D) treated PC3 cells, measured by MTT assays. **E.** Sphere assays in PC3 cells transfected with 3 different c-MYC siRNAs (20 nM). Cells were plated in six-well plate at indicated cell numbers and spheres scored on day 6. **F.** WB of cyclin D1 and EGFR in PC3 cells expressing miR-199a-3p (15 nM or 30 nM; 72 h). **G.** Luciferase assays showing the activity of WT or mutant cyclin D1 or EGFR 3-UTR in PC3 cells expressing miR-199a-3p. **H.** mTOR, phosphorylated AKT and AKT were determined by WB in DU145 cells treated with NC or miR-199a-3p (10 nM, 72 h). The expression levels of proteins in A, F and H were quantified by densitometry and normalized to the corresponding β-actin levels. Error bars in B, C-E, and G represent the SEM of three independent experiments. *P<0.05; **P<0.01. **I.** A schematic summarizing downstream targets of miR-199a-3p in PCa cells. CD44, MYC, cyclin D1, and EGFR (red) represent findings in this study whereas MET, PAK4, GSK3β, DNMT3A are targets reported in other studies.

When we transfected miR-199a-3p oligos into LAPC9 and PC3 cells, endogenous c-MYC protein levels decreased (Figure 7A; lanes 2 and 4 vs. lanes 1 and 3, respectively). Interestingly, exogenous miR-199a-3p did not significantly suppress the endogenous c-MYC protein levels in DU145 cells (Figure 7A), suggesting that c-MYC may not be the primary mediator of the miR-199a-3p effects in DU145 cells. Of note, miR-199a-3p downregulated exogenous c-MYC protein derived from a c-MYC-encoding cDNA construct in both PC3 and DU145 cells (Figure 7A; lanes 6 and 10 vs. lanes 5 and 9, respectively), suggesting that miR-199a-3p might target c-MYC coding sequence. Indeed, we identified a potential miR-199a-3p binding site in the c-MYC CDS (Supplementary Figure 3A). Further luciferase reporter assays confirmed that miR-199a-3p partially targeted c-MYC in PC3 cells (Figure 7B). Notably, mutation in the 3′-UTR of the miR-199a-3p binding site also restored luciferase activities to levels higher than in cells transfected with the WT 3-‘-UTR construct (Figure 7B). Consistent with c-MYC representing a functional downstream target of miR-199a-3p, knocking down endogenous c-MYC using 3 individual c-MYC-targeting siRNAs (Supplementary Figure 3B) or inhibiting c-MYC expression using JQ1 [36] (Supplementary Figure 3C) both inhibited PC3 cell viability (Figure 7C-7D). JQ1 and c-MYC siRNAs also inhibited clonogenic and sphere-forming capacities in PC3 cells, respectively (Figure 7E; Supplementary Figure 3D). These results indicate that reduced expression of c-MYC facilitates the inhibitory effect of miR-199a-3p in PCa cells such as PC3. Of note, either overexpression (data not sown) or knockdown of c-MYC (Supplementary Figure 3E–3F) had no effect on miR-199a-3p expression, although c-MYC was reported to modulate the expression of a number of miRNAs involved in the cell cycle and apoptosis [11, 37]. Collectively, these results suggest that c-MYC is regulated by miR-199a-3p in certain PCa cells.

Similar to c-MYC, miR-199a-3p also reduced the protein levels of cyclin D1 and EGFR in PC3 cells (Figure 7F). *In silico* analysis identified a putative miR-199a-3p binding site at the 3′-UTR of *CCND1* and *EGFR*, respectively (Supplementary Figure 3A). Luciferase reporter assays showed reduced luciferase activities in PC3 cells co-transfected with miR-199a-3p oligos and WT but not mutant cyclin D1 or EGFR 3′-UTR construct (Figure 7G). These results indicate that miR-199a-3p regulates cyclin D1 and EGFR expression in PC3 cells.

Finally, consistent with previous reports that miR-199a-3p also targets other oncogenic molecules such as mTOR [18, 20], we observed reduced mTOR protein levels in DU145 cells transfected with miR-199a-3p oligos (Figure 7H). Accompanying the mTOR reduction, pAKT was reduced without a decrease in the total AKT levels (Figure 7H).

The present study represents the very first comprehensive investigation on the biological functions of miR-199a-3p in PCa. We have shown that overexpression of miR-199a-3p greatly inhibits proliferation and clonal and sphere-forming capacities of CD44^+^ as well as bulk PCa cells. Importantly, similar inhibitory effects have also been observed in primary patient tumor-derived HPCa cells. Impressively, miR-199a-3p expression inhibits both tumor initiation and tumor growth in several PCa xenograft models. Preliminary studies have also revealed therapeutic efficacy of miR-199a-3p in retarding the growth of established xenograft tumors. Mechanistically, we find that like miR-34a, which is also under-expressed in CD44^+^ PCSCs [9], miR-199a-3p directly targets CD44 in several PCa cell types. The fact that 3 tumor-suppressive miRNAs, i.e., miR-34a [9], miR-708 [28], and miR-199a-3p (this study), simultaneously target 5 different sites at the *CD44* 3′-UTR (Figure 6A), highlights the critical importance of CD44 in regulating CSC properties [6–10]. Notably, our present study has provided evidence that miR-199a-3p may also exert tumor-suppressive functions via modulating several novel targets, i.e., c-MYC, cyclin D1, and EGFR. It seems that miR-199a-3p may target a different cohort of molecules in different PCa cell types. For example, in PC3 cells it downregulates CD44, c-MYC, cyclin D1 and EGFR whereas in DU145 cells it targets CD44 and mTOR. Regardless, by simultaneously targeting a cohort of pro-oncogenic molecules, miR-199a-3p manifests powerful PCa-suppressing effects, mainly through inhibiting cell proliferation (Figure 7I). Altogether, results presented herein provide a rational for developing miR-199a-3p into anti-PCa replacement therapeutics.

## MATERIALS AND METHODS

### Cells, xenografts, animals, reagents and antibodies

DU145, PC3, and PPC-1 cells were obtained from ATCC (Manassas, VA) and cultured in RPMI1640 medium whereas LAPC9 and VCaP cells were maintained in xenograft tumors. These cell line and xenograft models have been routinely utilized in our lab [6–10, 15, 24, 25, 38] and regularly authenticated by our institutional CCSG Cell Line Characterization Core using short tandem repeat (STR) analysis and checked to be free of mycoplasma contamination using the Agilent (Santa Clara, CA) MycoSensor QPCR Assay Kit (cat.#302107). NOD/SCID mice are produced mostly from our breeding colonies and purchased occasionally from the Jackson Laboratories (Bar Harbor). CD44^+^ cells were purified either by fluorescence-activated cell sorting (FACS) or magnetic-activated cell sorting (MACS). Antibodies used (Supplementary Table 1) included FITC- or PE-conjugated mouse anti-human CD44 used in FACS purification of CD44^+/hi^ PCa cells and a rabbit monoclonal ani-CD44 used in WB. Other reagents included FcR (130-059-901, Miltenyi Biotec), anti-FITC microbeads (120-000-293, Miltenyi Biotec) and anti-PE microbeads (120-000-294, Miltenyi Biotec).

### Xenograft tumor and primary human PCa (HPCa) processing

Xenograft tumor processing has been described previously [6–10] and detailed in ref. [25]. All HPCa samples were obtained with the written informed patients' consent from Da Vinci robotic surgery in accordance with federal and institutional guidelines with the approved IRB protocol (MDACC LAB04-0498) and processed as described [9, 39] with minor modifications. Briefly, tumor pieces were trimmed, cut into small chunks and rinsed with cold PBS twice. Tumor pieces were then digested in the order of collagenase/dispase solution (Collagenase, 17018-029, GIBCO, Life Technology; Dispase, 17105-041, GIBCO, Life Technology) at 37°C incubator under rotating conditions for 8~12 h, 0.05% trypsin/EDTA for 5 min and DNase I for 5 min. Samples were triturated through 20 G needles and cells filtered through a 100-μm cell strainer. After removing the red blood cells, cell suspension was filtered through a 40-μm cell strainer and collected in WIT media (01-0009-500, Stemgent, San Diego, CA). These cells were used as bulk HPCa cells. CD44^+^ HPCa cells were obtained by sorting TROP2^+^CD44^+^ cells from freshly prepared bulk cells [39]. Antibodies used herein (Supplementary Table 1) included mouse IgG2a APC (allophycocyanin) isotype control, APC-conjugated anti-human TROP-2 monoclonal antibody, and PE-conjugated mouse anti-human CD44 or mouse IgG2b.

### Transfection and lentiviral infection

In general, bulk or freshly purified CD44^+^ PCa cells and HPCa cells were transfected with NC miRNA or miR-199a-3p mimics (3p) using lipofectamine RNAiMAX (Invitrogen, Life Technology). In some experiments, bulk or purified CD44^+^ cells were infected with empty (pGIPZ-Ctrl) or miR-199a-3p expressing lentivirus (pGIPZ-199A) at MOI (multiplicity of infection) of 10-20 for 72 h. pGIPZ-199A vector was established from the backbone of GIPZ lentiviral shRNA (GIPZ-Ctrl) (GE Dharmacon), in which pre-miR-199A1 and its frank sequences were cloned into XhoI and MluI sites (Supplementary Figure 1A). For therapeutic experiments, PCa cells were infected with lenti-Ctrl or lenti-199A1 at MOI of 10-20 for 48 h followed by puromycin selection. Lenti-199A1 was constructed from the backbone of TRIPZ inducible lentiviral shRNA (lenti-Ctrl) (GE Dharmacon). The same sequence as in pGIPZ-199A was cloned into ClaI and MluI sites of TRIPZ inducible shRNA (Supplementary Figure 1A).

### Tumor regeneration and therapeutic experiments

Tumor transplantations were performed as previously described [9, 25]. For subcutaneous tumor experiments, we usually do two injections per mouse, 5 - 10 animals per group. For therapeutic experiments, DU145 cells were infected with negative control (lenti-Ctrl) and miR-199a-3p lentivirus (lenti-199A) and subcutaneously implanted into NOD/SCID female mice. When tumors became palpable, two groups of mice were randomly divided into two subgroups, each one of which was administrated with the doxycycline in the food (2 μg). Tumor volume was then measured every 2 - 3 days for approximately two months.

### Real-time reverse transcription-polymerase chain reaction (RT-qPCR) and western blotting

In brief, total RNA was extracted from unsorted or purified CD44^+^ and CD44^−^ PCa cells by using the mirVana™ miRNA Isolation Kit (P/N: 1560, Ambion, Austin, TX). cDNA was synthesized using 10 ng of total RNA and RT primers for RNU48, the internal “housekeeping” miRNA control or for miR-199a-3p. qPCR was performed using the synthesized cDNA, and RNU48 or miR-199a-3p miRNA primers (Ambion, Life Technology). We first processed the raw data using the ΔCt method, by which the expression level of miR-199a-3p in each sample was normalized to that of RNU48. We then compared the relative expression levels of miR-199a-3p (and/or miR-199a-5p) in different experimental groups (e.g., CD44^+^ vs. CD44^−^ cell, NC vs. miR-199a-3p, lenti-Ctrl vs. lenti-199A1, etc) by normalizing to the corresponding CD44^−^, NC, or Ctrl group (which was considered as 1). Western Blotting was routinely performed using primary antibodies listed in Supplementary Table 1, ECL Mouse IgG, HRP-Linked whole Ab (NA931V, GE Healthcare Life Sciences), ECL Rabbit IgG, and HRP-Linked Whole Ab (NA934V, GE Healthcare Life Sciences)

### Immunohistochemistry (IHC)

Briefly, formalin-fixed paraffin-embedded tissue sections (4 μm) were deparaffinized and hydrated in xylene followed by graded alcohols to water. Endogenous peroxidase activity were blocked with 3% H_2_O_2_ for 10 min. After antigen retrieval in 10 mM Citrate Buffer (pH 6.0), non-specific binding was blocked by Background Sniper (BS966H, Biocare Medical) and slides were incubated with CD44, Ki-67, or lamin A antibodies (Supplementary Table 1) at 1:100 dilution at 4°C overnight. Next day, slides were thoroughly washed and visualized upon incubation with polymer-conjugated horseradish peroxidase and Sigma Tablet DAB.

### Clonal, sphere-formation and Matrigel-based clonogenic assays

For holoclone assays, cultured PCa or HPCa cells were plated at 500 ~ 5000 cells per well in six-well plates and the number of colonies enumerated in 1-2 weeks upon crystal violet staining. For sphere-formation assays, PCa cells were plated at 500 ~ 5000 cells per well in ultra-low attachment plates and cultured in WIT medium for 2-3 weeks followed by determining the number of colonies under a microscope. For Matrigel-based clonogenic assays, a mixture of 40 μl of medium with 500-5,000 cells and 40 μl of Matrigel solution were seeded along the edge of the wells in 24-well plates followed by counting the number of colonies in 2 - 3 weeks.

### BrdU incorporation assays and cell cycle analysis

For BrdU incorporation assays, cells plated on coverslips one day before were pulsed for 3 - 4 h with 10 μM BrdU (B5002, Sigma), fixed in 4% paraformaldehyde and incubated with mouse anti-human BrdU (B2531, Sigma) antibody at 4°C overnight. After thorough washing, coverslips were incubated at room temperature for 1h with secondary antibody, i.e., Alexa Flour 594-conjugated goat anti-mouse IgG (1:500). Coverslips were then counterstained with DAPI (1:1000) and mounted with 10 μl Gold Antifade Reagent (936590, Prolong). Images were acquired under microscope (Nikon, Eclipse E800). For cell cycle analysis, 48 h after transfection when cells reached approximately 60-80% confluence, cells were harvested and fixed in cold 70% ethanol and incubated in propidium iodide (PI) solution, with 20 μg/ml PI, 50 μg/ml RNase A, 0.02% NP40 in PBS at 4 °C for 30 min and then used for DNA content analysis.

### Site-specific mutagenesis and luciferase assays

The luciferase reporter (pMIR-REPORT, Ambion) carrying the wild-type (WT) human *CD44* 3′-UTR fragment was described previously [9]. Specifically, the human *CD44* 3′-UTR was amplified and cloned into SacI and HindIII of pMIR-REPORT (Supplementary Table 2). Mutant *CD44* 3′-UTR construct was performed using QuickChange II Site-Directed Mutagenesis Kit (Agilent Technologies) and primers in Supplementary Table 2. *Cyclin D1*, *EGFR* and *MYC* 3′-UTR wild type (WT) and mutant (MUT) sequences (Supplementary Table 2) were synthesized by Sangon Biotech (Shanghai, China) and inserted into Xba I site of pGL3-basic vector (Promega). For luciferase assays [9, 40], 150 ng of WT or mutant plasmid was co-transfected with 5 nmol of miRNA oligos and 1 ng of *Renilla* luciferase plasmid (phRL-CMV) for 48 h and the relative *Firefly* and *Renilla* luciferase activities determined by Dual-Luciferase Assay Kit (Promega).

### Statistical analysis

In general, statistical differences and variances for cell number, percentage of CD44^+^ cells, DNA content, sphere/cloning efficiency and tumor weights, etc. were determined by Student's *t*-test. The Fisher's exact and χ^2^ tests were used to compare tumor incidence. All results were presented as mean ± S.D or mean ± SEM. P < 0.05 was considered statistically significant.

## SUPPLEMENTARY MATERIALS FIGURES AND TABLES


