# Supplementary material for: miR-199a-3p targets stemness-related and mitogenic signaling pathways to suppress the expansion and tumorigenic capabilities of prostate cancer stem cells
Source: Oncotarget. 2016 Jul 18;7(35):56628–42. doi: 10.18632/oncotarget.10652 (PMC5302940; doi:10.18632/oncotarget.10652)
Supplement: Supplementary file 1 [file oncotarget-07-56628-s001.pdf]

miR-199a-3p targets stemness-related and mitogenic signaling pathways to suppress the expansion and tumorigenic capabilities of prostate cancer stem cells

SUPPLEMENTARY FIGURES AND TABLES

A Inserted sequence in lentivirus (pGIPZ-199A and lenti-199A):

5'- CAGCTAATGCCACATCTGGAACTGTTTACAGTGCGATTCCGCCGAGAAATCAGTGGCCGCCTTCCT
GGTGCTGCCGGCACGCACGCGTGCGCGCGCGCACACACACACACACGTGTGTGTGCGCC
CCTCCTCCCCACCCCCGACCCCCAAAGAGTCAGACATTCCTCCTGAGCCCAGAAGCCACGATCCC
AAACCCTGCCTCCTGCTCCGCCTCCCCCACTCTTTAGGATTTCCTGAAAACCCAGAACTTTCTCCA
GATGCGAGCCGGCCCCAGCCTTGCCACGTCAGAAGGGACAGAGCGGATCGTCTCGGGAAGAGTG
GTGGTTTCCTTGGCTGCTCAGAGGTGCTGAGGCACGGCCTGGCCTGGTGGCCCCAGCGTCTGCC
TGGGGGGTTCTGCAGGATGGATAGCCGGCCCCGCCAACCCAGTGTTCAGACTACCTGTTCAGGAG
pre-miR-199A1
GCTCTCAATGTGTACAGTAGTCTGCACATTGGTTAGGCTGGGCTTGGGTGAGCGGCTCGTCGAGAC
hsa-miR-199a-3p
AGGCCCCCCAAACTCGCCGGCAGGTGAGTGTCATTTTCCACCACCCCGTTTCCCACTGTGGCAGA
GCCTCGCATAGAAGATTCGAGGGCCTGGGTGGGAGAAGAGGCACTGGGGAAAGGCAGAGGGAGC
CCCGAGGCCGGCCGAGGGGTTGGAGGCCCTGGCCCTATCAGTCTGCCATCCCCACCCCATGGCT
GTAAATGTCTTGTTTATTTTTTAAATAAAGAGATATTGATGTCTTGTGTCTCACTGAGGCATCTAAGAG
GGTGGCCTCCTCCTCAGGGGTGGCTGCCACGTGTGTGACTCCAAGGGTCCCTGCTGGGATCCTA
ACTTGG-3'

	Forword Primer	Reverse Primer
pGIPZ-199A	(XhoI)TACTCGAGCAGCTAATGCCACATC	ATACGCGTCCAAGTTAGGATCCCA(MluI)
lenti-199A	(ClaI) TAATCGATCAGCTAATGCCACATC	ATACGCGTCCAAGTTAGGATCCCA(MluI)

B

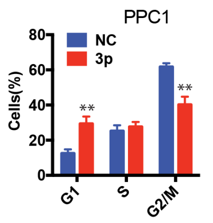

C

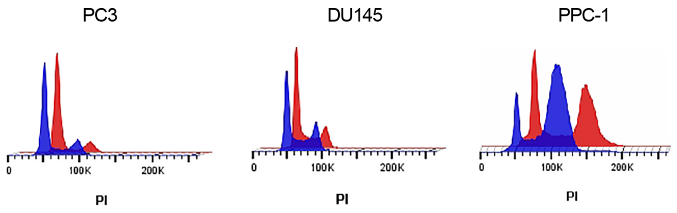

D

		G1	S	G2/M
PC3	NC	59.5	22.7	15.7
	3p	66.7	14.8	17.3
DU145	NC	42.3	32.7	23.7
	3p	46.7	34.1	17.7
PPC-1	NC	11.5	25.5	61.7
	3p	22.8	25.9	49.1

E

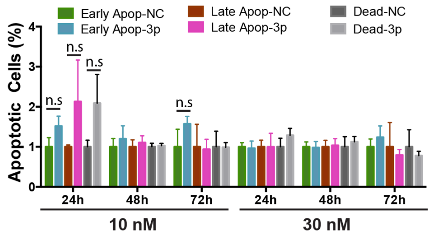

Supplementary Figure 1: miR-199a-3p genomic loci and its effect on apoptosis in PCa cells. A. The pre-miR-199A1 sequence inserted in either the pGIPZ-199A or lenti-199A lentiviral vectors established in our lab. The PCR primers that harbor the cloning restriction sites are indicated below. **B-D.** miR-199a-3p effects on cell-cycle profiles in the 3 PCa cell types. (B) DNA content analysis in miR-199a-3p or NC transfected PPC-1 cells. Bars represent the average % of cells in each cell-cycle phase (n=3 independent experiments). (C) Representative flow histograms showing the effects of miR-199a-3p on cell-cycle profiles in the 3 PCa cell types. (D) Table summarizing the mean % of cells in different cell-cycle phases in NC and miR-199a-3p transfected cells. **E.** DU145 cells transfected with miR-199a-3p or NC (30 nM) at different concentrations were harvested at 24-72 h and used in FITC-Annexin V and PI analysis. Early apoptosis: Annexin V positive/PI negative; Late apoptosis: Annexin V positive/PI positive; Dead (late necrotic) cells: PI positive only. n.s, not statistically significant (n=3 independent experiments). All bars and data points represent the mean ± S.D; **P<0.01.

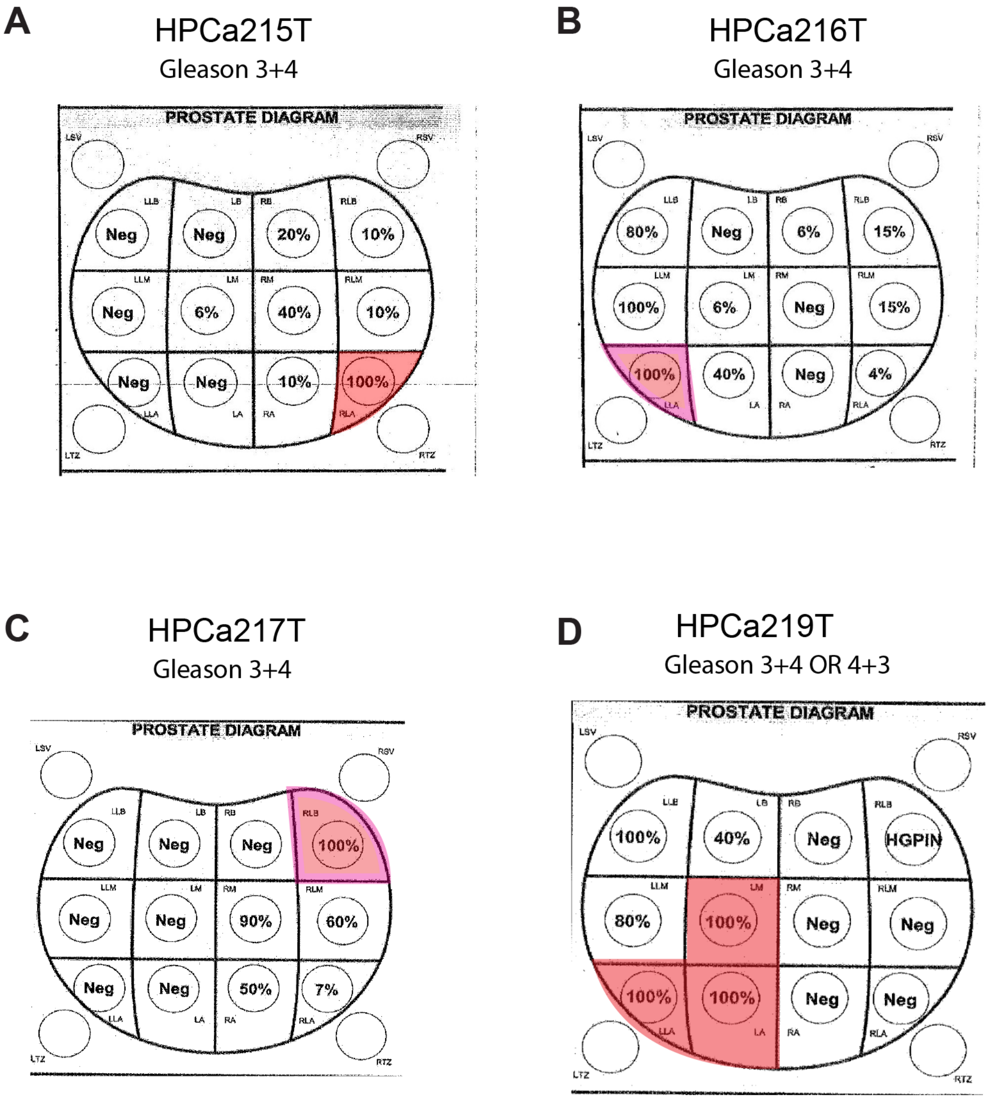

Supplementary Figure 2. Biopsy reports of HPCa samples and transfection efficiency in HPCa cells. A-D. Biopsy reports of HPCa215, HPCa216, HPCa217 and HPCa219 patient tumor samples. Red areas indicate the areas with ~100% tumor involvement used in present study.

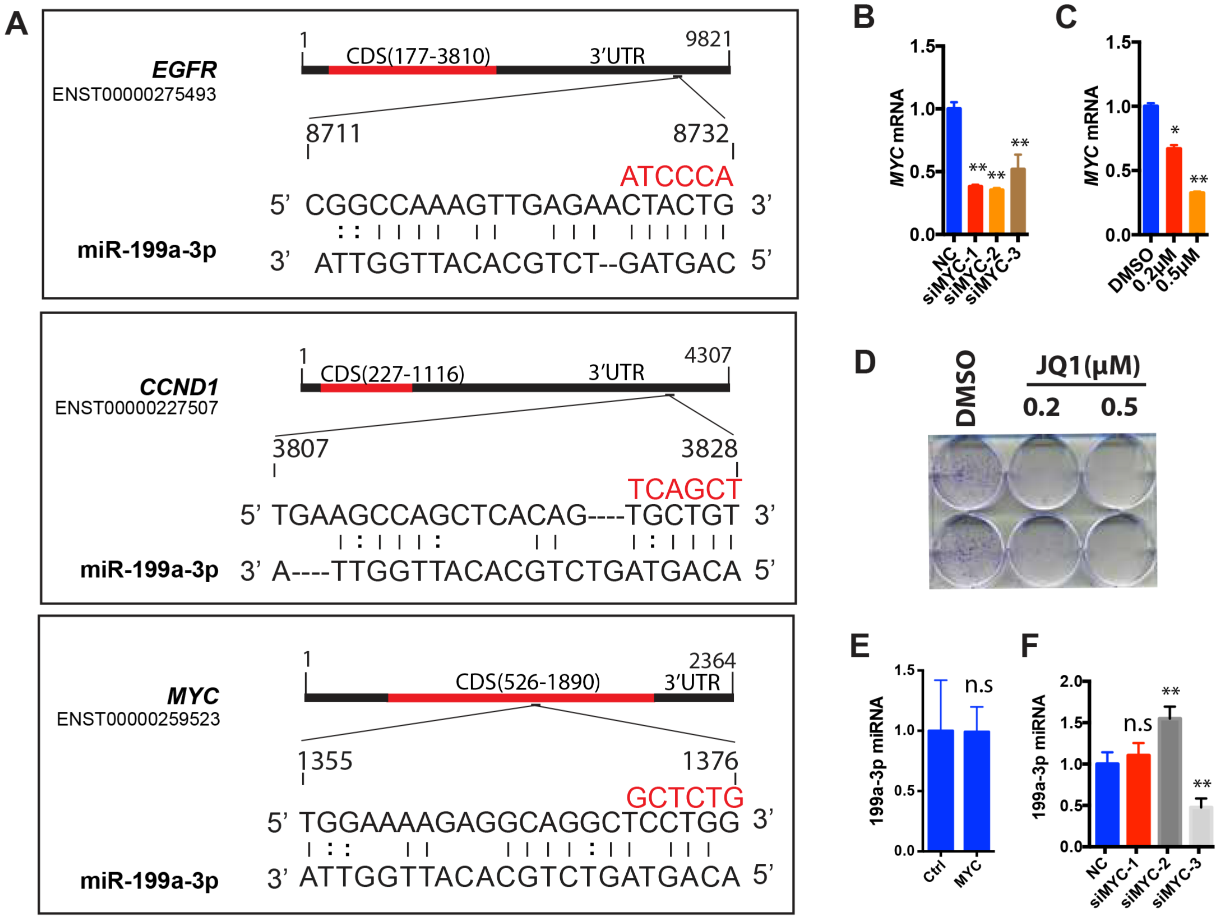

Supplementary Figure 3. c-Myc, cyclin D1 and EGFR as novel miR-199a-3p targets. A. Schematic of the predicted (by RNA22 program) binding site of miR-199a-3p at the *CCND1* and *EGFR* 3'-UTRs or *c-MYC* CDS. The nucleotides in red indicate the mutant sequences used in Fig. 7B and Fig. 7G. **B.** RT-qPCR analysis of the knockdown efficiency of c-Myc siRNA-1, 2 and 3 in PC3 cells after 72 h transfection (10 nM). **C.** c-Myc mRNA levels decreased as determined by RT-qPCR in PC3 cells treated with JQ1 for 72 h. **D.** Holoclone assays in PC3 cells with indicated JQ1 treatment. 1,000 cells were plated and the image was taken on day 7. **E-F.** miR-199a-3p mRNA levels in PC3 cells transfected with c-Myc expression vector (E) or c-Myc siRNA-1,2, and 3 (F) for 72 h. The relative miR-199a-3p levels were first normalized to the respective RNU48 levels in each sample and then normalized to the corresponding Ctrl (E) or NC (F) group (as 1). Bars in (B-C) and (E-F) are the mean ± S.D representative of three independent experiments with 3 replicate in each condition. $^{*}P<0.05$; $^{**}P<0.01$; n.s, not statistically significant.

Supplementary Table 1: Antibodies used in the current study

Antibody	Source	Type	Catalog#	Company	Remarks	Usage
AKT	Rabbit	Monoclonal	4691	Cell Signaling	Pan-AKT antibody	WB
CD44	Mouse	Monoclonal	555478	BD Pharmingen	Clone G44-26; FITC-conjugated	FACS
CD44	Mouse	Monoclonal	555479	BD Pharmingen	Clone G44-26; PE-conjugated	FACS
CD44	Mouse	Monoclonal	550329	BD Pharmingen	Clone G44-26; recognizes 80-95KD	IHC
CD44	Rabbit	Monoclonal	ab51037	abcam	Clone EPR1013Y	WB
Cyclin D1	Rabbit	Monoclonal	ab134175	abcam	Clone EPR2241	WB
EGFR	Rabbit	Monoclonal	4267	Cell Signaling		WB
IgG2a	Mouse	Monoclonal	IC003A	R&D System	Clone 20102, APC-conjugated	
IgG2b	Mouse	Monoclonal	555742	BD Pharmingen	Isotype control, FITC conjugated	FACS
IgG2b	Mouse	Monoclonal	555743	BD Pharmingen	Isotype control, PE conjugated	FACS
Ki-67	Mouse	Monoclonal	M7240	Dako	Clone M1B-1	IHC
Lamin A	Rabbit	Polyclonal	2035	Cell Signaling		IHC
mTOR	Rabbit	Monoclonal	5536	Cell Signaling		WB
MYC	Rabbit	Monoclonal	ab32072	abcam	Clone Y69	WB
phospho-Akt	Rabbit	Monoclonal	4060	Cell Signaling	Recognizes Ser473	WB
TROP-2	Mouse	Monoclonal	FAB650A	R&D System	Clone 77220, APC-conjugated	FACS
β-actin	Mouse	Monoclonal	A1978	Sigma-Aldrich	Clone AC-15	WB

Supplementary Table 2: Primers and inserted sequences used for luciferase-reporter plasmids

**CD44 3' UTR-WT**	Forward Primer: AGAGCTCCACCTACACCATTATCTTG<br>Reverse Primer: TAAGCTTGGAAGTCTTCAGGAGACAC
**CD44 3' UTR-MUT**	Forward Primer: CTTAACAGATGCAATGTG*CctgTc*ATTGTTTCATTGCGAATC<br>Reverse Primer: GATTCGCAATGAAACAAT*gAcAgG*CACATTGCATCTGTTAAG
**cyclin D1 3' UTR-WT**	TAATCTGTTATGTACTAGTGTTCTGTTTGT<br>TATTGTTTTGTTAATTACACCATAATGCTAATT<br>TAAAGAGACTCCAAATCTCAATGAAGCCA<br>GCTCACAG*TGCTGT*GTGCCCCGGTCACCTAGC<br>AAGCTGCCGAACCAAAAGAATTTGCACCCCGCTGCGGGCCCACGTG<br>GTTGGGGCCCTGCCCTGGCAGGGTCATCCTGTGCTCGG
**cyclin D1 3' UTR-MUT**	TAATCTGTTATGTACTAGTGTTCTGTTTGTTATTG<br>TTTTGTTAATTACACCATAATGCTAATTT<br>AAAGAGACTCCAAATCTCAATGAAGCCAGCTC<br>ACAG*TCAGCT*GTGCCCCGGTCACCT<br>AGCAAGCTGCCGAACCAAAAGAATTT<br>GCACCCCGCTGCGGGCCCACGTGGTTGGGGC<br>CCTGCCCTGGCAGGGTCATCCTGTGCTCGG
**EGFR 3' UTR-WT**	ACCTCAGACCGATTAAACGCAAATCTCTGGGGCTGAAACCCAAGCATTCGTAG<br>TTTTTAAAGCTCCTGAGGTCATTCCAATGTGCGGCCA<br>AAGTTGAGAA*CTACTG*GCCTAGGG<br>ATTAGCCACAAGGACATGGACTTGGAGGCAAATTCTGCAGGTGTATGTG<br>ATTCTCAGGCCTAGAGAGCTAAGACACAAAGACCTCCACATCTG
**EGFR 3' UTR-MUT**	ACCTCAGACCGATTAAACGCAAATCTCTGGGGCTGAAACCCAAGCATT<br>CGTAGTTTTTAAAGCTCCTGAGGTCATTCCAATGTGCGGCCAAA<br>GTTGAGAA*ATCCCA*GCCTA<br>GGGATTAGCCACAAGGACATGGACTTGGAGGCA<br>AATTCTGCAGGTGTATGTGATTCTCAGGCC<br>TAGAGAGCTAAGACACAAAGACCTCCACATCTG
**MYC 3' UTR-WT**	TGCTCCATGAGGAGACACCGCCCACCACCAGC<br>AGCGACTCTGAGGAGGAACAAGAAGATGAGGAAG<br>AAATCGATGTTGTTTCTGTGGAAAAGAGGCAGGC*TCCTGG*CAAAAGGTCAG<br>AGTCTGGATCACCTTCTGCTGGAGGCCACAGCAAACCTCCTCACAGCCCACT<br>GGTCCTCAAGAGGTGCCACGTCTCCACACATCAGCACA
**MYC 3' UTR-MUT**	TGCTCCATGAGGAGACACCGCCCACCAC<br>CAGCAGCGACTCTGAGGAGGAACAAGAAGATGA<br>GGAAGAAATCGATGTTGTTTCTGTGGAAAAGAGGC<br>AGGC*GCTCTG*CAAAAGGTCAG<br>AGTCTGGATCACCTTCTGCTGGAGGCCACAGC<br>AAACCTCCTCACAGCCCACTGGT<br>CCTCAAGAGGTGCCACGTCTCCACACATCAGCACA

Note: The seed sequence and mutant region were highlighted in red.
